# Supplementary figures and images for: Thymically-derived Foxp3+ regulatory T cells are the primary regulators of type 1 diabetes in the non-obese diabetic mouse model
Source: PLoS One. 2019 Oct 24;14(10):e0217728. doi: 10.1371/journal.pone.0217728 (PMC6812862; doi:10.1371/journal.pone.0217728)

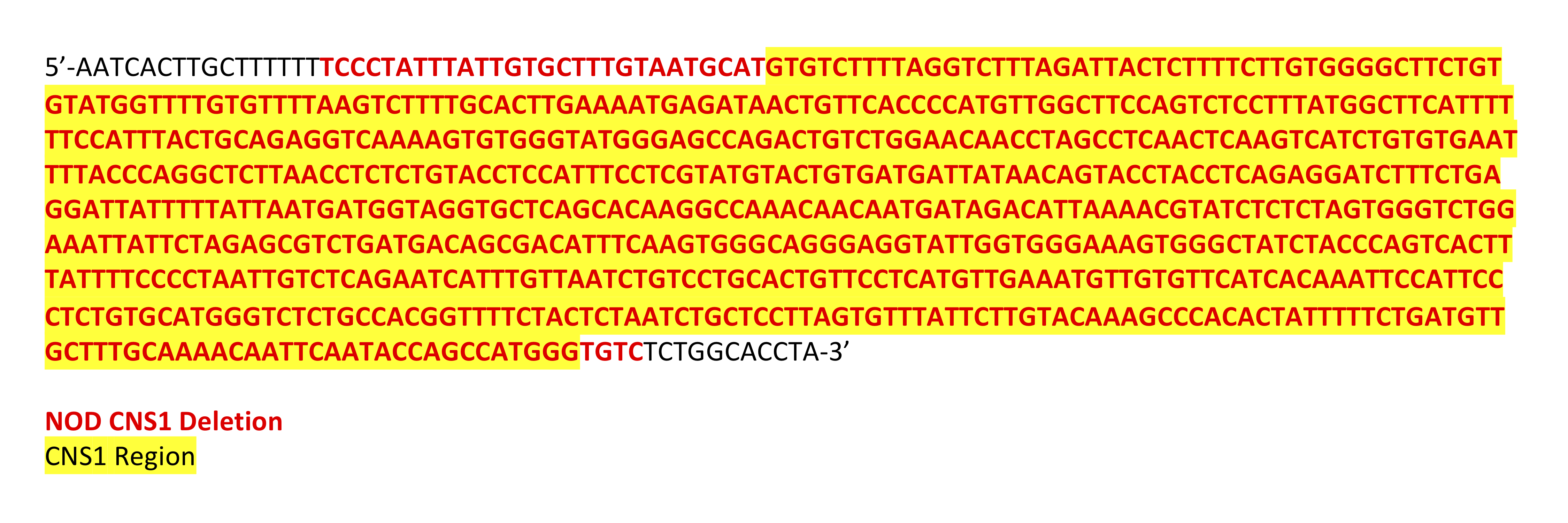

Supplement: S1 Fig — Displayed here is the +1960 to +2722 region from the transcriptional start site of Foxp3. The original CNS1 region identified in B6 mice (+2003 to +2707) is highlighted in yellow, while the sequence of the deleted region generated in the NOD background with CRISPR/Cas9 (+1976 to +2711) is bolded in red. (TIF) [file pone.0217728.s001.tif]

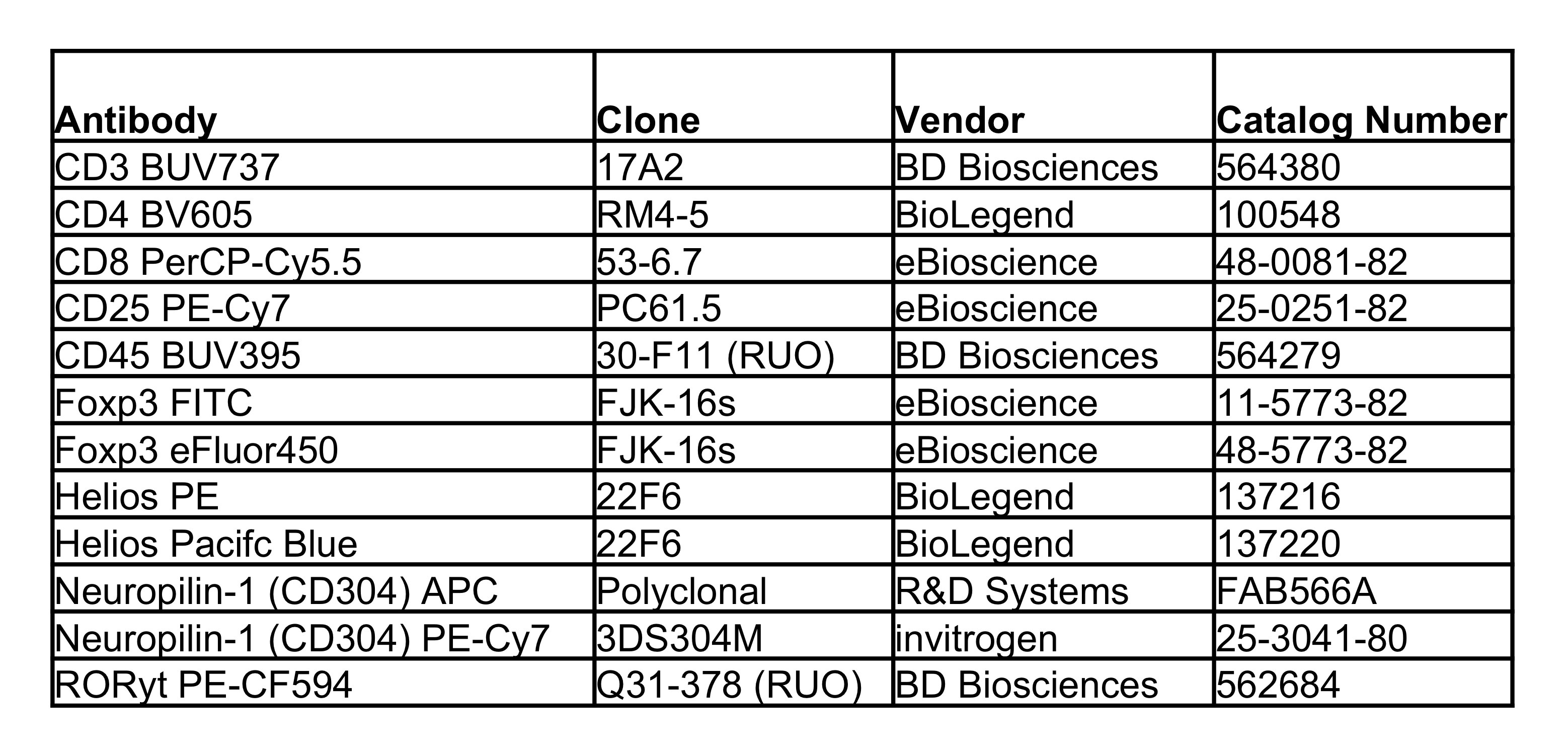

Supplement: S1 Table — (TIF) [file pone.0217728.s002.tif]
